# Biodistribution, Radiation Dosimetry, and Kinetic Modeling of [^18^F]FPEB PET Imaging of mGluR5 in Healthy Asian Subjects

**DOI:** 10.3390/ph19091375

**Published:** 2026-08-31

**Authors:** Wanying Xie, Sarah Beishan Tai, Chang-Tong Yang, Sidney W. K. Yu, Young Soon Tay, Ruban Poopalalingam, Darren Liang-Khai Koh, Kerry Riffel, Wenping Li, Chih-Liang Chin, Asad abu Bakar Ali, Darren Wan-Teck Lim, David Chee Eng Ng

**Affiliations:** 1 Department of Nuclear Medicine and Molecular Imaging, Radiological Sciences Division, Singapore General Hospital, Outram Road, Singapore 169608, Singapore; sarah.tai.beishan@singhealth.com.sg (S.B.T.); tay.young.soon@sgh.com.sg (Y.S.T.); david.ng.c.e@singhealth.com.sg (D.C.E.N.); 2 Duke-NUS Medical School, 8 College Road, Singapore 169857, Singapore; ruban.poopalalingam@singhealth.com.sg (R.P.); darren.lim.w.t@singhealth.com.sg (D.W.-T.L.); 3 Advanced Medicine Imaging Pte. Ltd., 1 Biopolis Drive, Singapore 138622, Singapore; 4 Department of Anaesthesiology, Singapore General Hospital, Singapore 169608, Singapore; 5 Merck & Co., Inc., West Point, PA 19486, USA; kerry_riffel@merck.com (K.R.); wenping_li@merck.com (W.L.); 6 Merck & Co., Inc., South San Francisco, CA 94080, USA; chih-liang.chin@merck.com; 7 Merck & Co., Inc., MSD International GmBH, Singapore 049910, Singapore; asad.abu.bakar.ali@msd.com; 8 Singhealth Investigational Medicine Unit, Singhealth, Singapore 168582, Singapore

**Keywords:** [^18^F]FPEB, radiation dosimetry, modeling, mGluR5, human brain

## Abstract

**Background:** Metabotropic glutamate receptor subtype 5 (mGluR5) is a promising therapeutic target for a range of neuropsychiatric disorders, and [^18^F]FPEB is a PET radioligand developed for imaging central mGluR5 receptor availability. This study aimed to determine the biodistribution, kinetic characteristics, and radiation dosimetry of [^18^F]FPEB in healthy Asian subjects and to evaluate the feasibility of reference tissue models as alternatives to conventional blood-based kinetic modeling. **Methods:** Five healthy Asian male volunteers underwent whole-body and dynamic brain PET imaging following administration of [^18^F]FPEB. Radiation dosimetry was estimated using OLINDA/EXM software. Tracer kinetics were analyzed using both blood-based compartmental models and reference tissue models to derive regional binding potentials (BPND) and volumes of distribution. Model performance was assessed using the Akaike Information Criterion (AIC). **Results:** The mean whole-body effective radiation dose was 0.703 mSv/mCi, with the gallbladder identified as the critical organ receiving the highest absorbed dose (5.63 mGy/mCi). Brain time–activity curves demonstrated the highest tracer uptake in the nucleus accumbens, followed by the cingulate gyrus, putamen, and insula, with moderate uptake in the thalamus and globus pallidus and the lowest uptake in the brainstem and cerebellum. Using the simplified reference tissue models (SRTM/SRTM2), the highest BPND values were observed in the nucleus accumbens, followed by the cingulate gyrus, putamen, and insula. In contrast, two-tissue compartment models (2TCM/2TCM-C) showed the highest BPND values in the hippocampus, followed by the amygdala, nucleus accumbens, and caudate nucleus. Although differences were observed in the absolute BPND values and regional ranking, both modeling approaches identified similar brain regions with high mGluR5 binding. **Conclusions:** [^18^F]FPEB demonstrated a favorable radiation dosimetry profile and supports the feasibility of reference tissue models for BPND estimation in healthy Asian subjects. These findings provide preliminary baseline data, although validation in larger cohorts is needed to confirm the pharmacokinetic characteristics and modeling approach.

## 1. Introduction

Excessive glutamatergic neurotransmission has been implicated in a broad range of neuropsychiatric disorders, highlighting the glutamatergic system as an important therapeutic target [1,2]. Among the glutamate receptor subtypes, metabotropic glutamate receptor type 5 (mGluR5) has attracted considerable interest because of its role as a postsynaptic G-protein-coupled receptor involved in the regulation of excitatory neurotransmission and synaptic plasticity [3,4]. Pharmacological inhibition or negative allosteric modulation of mGluR5 has demonstrated anxiolytic, antidepressant, and anti-addictive effects in preclinical studies, supporting its potential as a therapeutic target in major neuropsychiatric conditions, including anxiety disorders, major depressive disorder, and substance use disorders [5,6]. In addition, accumulating evidence implicates mGluR5 in the pathogenesis of several neurodegenerative disorders, including Alzheimer’s disease, amyotrophic lateral sclerosis, and multiple sclerosis, suggesting broader therapeutic applications for mGluR5 modulators [7,8,9].

Despite promising preclinical findings, the clinical translation of mGluR5-targeted therapeutics has been challenging. Several candidate compounds, including basimglurant and fenobam, have demonstrated limited clinical efficacy and/or undesirable adverse effects in human studies [10,11,12]. These disappointing outcomes may reflect discrepancies between preclinical models and human disease biology, resulting in differences in target engagement, pharmacokinetics, therapeutic windows, and safety profiles [13,14]. Drug development for central nervous system (CNS) disorders is further complicated by the requirement for adequate blood–brain barrier penetration, making systemic pharmacokinetic measurements poor surrogates of intracerebral drug exposure [15]. Although cerebrospinal fluid sampling can provide additional information, it remains invasive and does not necessarily reflect regional brain tissue concentrations [16]. Consequently, non-invasive methods capable of quantifying target engagement and brain pharmacokinetics in vivo are essential for the efficient development of CNS therapeutics. Positron emission tomography (PET) offers a sensitive and quantitative approach for assessing receptor availability and target occupancy in humans, thereby facilitating drug selection, dose optimization, and early decision-making in CNS drug development [16,17,18].

Among the available PET radioligands for imaging mGluR5, (^18^F)-3-fluoro-5-[(pyridin-3-yl)ethynyl]benzonitrile ([^18^F]FPEB) has emerged as one of the most promising tracers because of its favorable physical half-life and high affinity and specificity for mGluR5 [19,20]. Nevertheless, relatively few human pharmacokinetic studies of [^18^F]FPEB have been reported, and most have been conducted in US populations [21,22]. Furthermore, quantitative analyses have largely relied on arterial blood sampling, which is invasive, labor-intensive, and susceptible to procedural variability due to the need for precise blood collection and metabolite correction procedures [23]. Therefore, a robust, reproducible, and clinically practical method for quantifying mGluR5 availability in humans remains an unmet need. Previous studies have suggested that the cerebellar cortex [24,25] and pons [22] exhibit negligible or minimal specific [^18^F]FPEB binding, indicating their potential utility as reference regions for non-invasive kinetic modeling. Validation of such reference tissue approaches could facilitate wider clinical implementation by eliminating the need for arterial blood sampling while maintaining quantitative accuracy.

In the present study, we evaluated the biodistribution, radiation dosimetry, and pharmacokinetics of [^18^F]FPEB in healthy Asian volunteers and characterized the regional distribution of mGluR5 in the human brain. Given the limited availability of [^18^F]FPEB imaging data in Asian populations, this study also provides important population-specific pharmacokinetic and dosimetry information. In addition, we investigated the feasibility of reference tissue modeling using candidate reference regions with minimal mGluR5 expression and compared the resulting pharmacokinetic parameters with those obtained using arterial input function-based analyses. We hypothesized that cerebellar cortex- and brainstem-based reference tissue models would provide pharmacokinetic estimates comparable to those derived from arterial blood sampling, thereby supporting a simplified and less invasive approach for quantitative mGluR5 PET imaging.

## 2. Results

### 2.1. Part I: Whole-Body Biodistribution and Radiation Dosimetry

#### 2.1.1. Biodistribution

Five healthy male subjects (mean age 38 ± 7 years; range 28–47 years) underwent whole-body PET imaging following administration of [^18^F]FPEB (4.5 ± 0.5 mCi).

**Figure 1:**
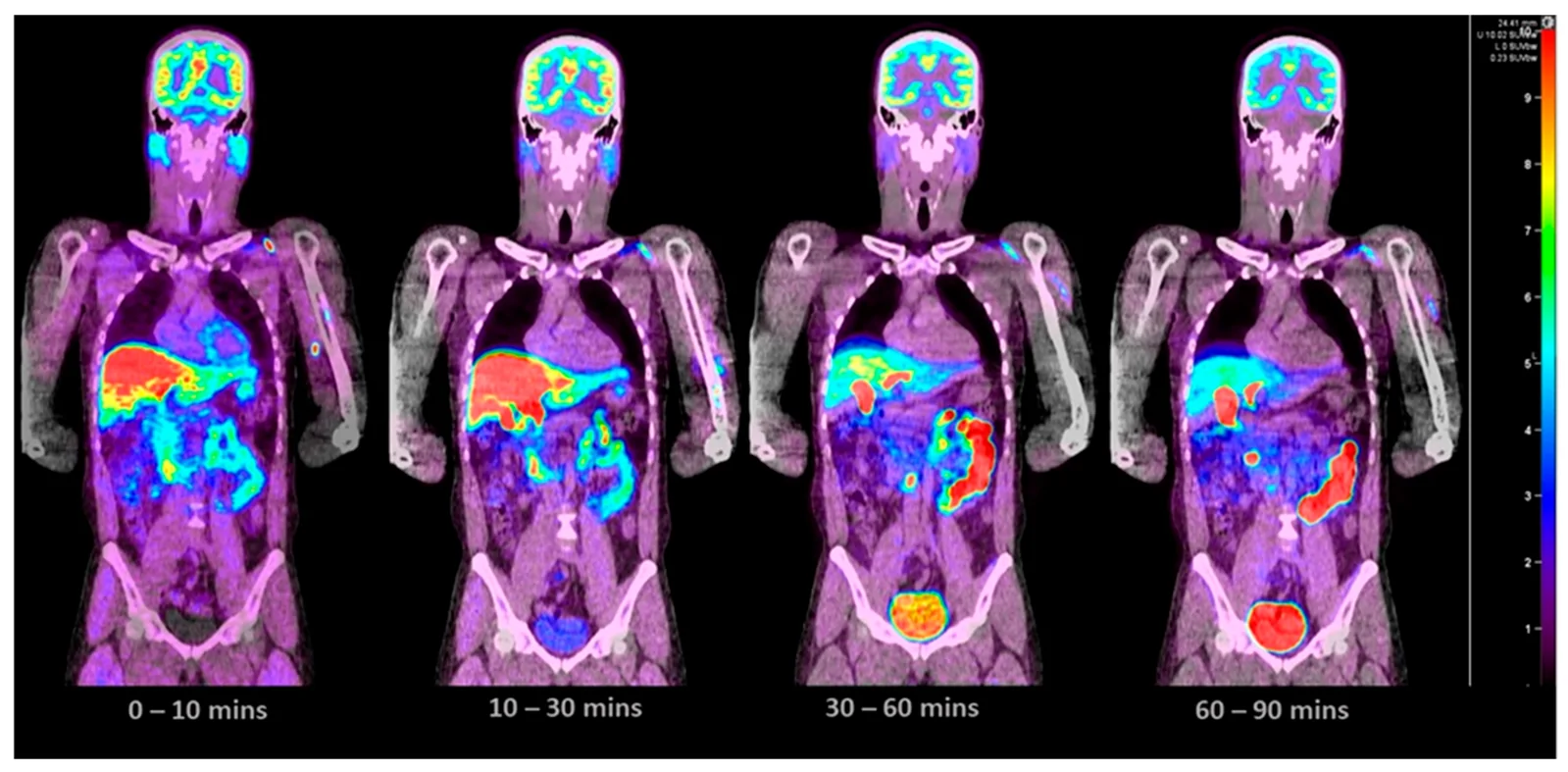
Sequential whole-body PET-CT images of a representative subject demonstrating biodistribution of [^18^F]FPEB. Rapid [^18^F]FPEB uptake is noted in the brain. Prompt [^18^F]FPEB tracer excretion is noted via both the hepatobiliary (as evidenced by tracer transit through the liver, followed by bowel) and the urinary system (as evidenced by urinary bladder tracer accumulation).

[^18^F]FPEB demonstrated rapid uptake in the brain, which was evident on the first whole-body scan acquired approximately 10 min after injection. High cerebral activity was maintained throughout the 60 min imaging period. Tracer clearance occurred predominantly through hepatobiliary and renal pathways. Within the first hour post-injection, substantial activity was observed in the liver, gallbladder, intestines, kidneys and urinary bladder. Progressive accumulation of radioactivity in the urinary bladder was observed from approximately 30 min post-injection onwards, indicating renal excretion of the tracer and/or its radiometabolites (Figure 1).

#### 2.1.2. Radiation Dosimetry

Source organ residence times for all subjects are summarized in Table 1. Radiation absorbed dose estimates and effective doses calculated using OLINDA/EXM are presented in Table 2.

The gallbladder wall received the highest absorbed radiation dose, with a mean value of 5.63 mGy/mCi. The mean whole-body effective dose was 0.66 mSv/mCi.

#### 2.1.3. Safety

No adverse events, clinically significant laboratory abnormalities, or abnormal physical examination findings were observed following administration of [^18^F]FPEB.

### 2.2. Part II: Brain Pharmacokinetics

#### 2.2.1. Plasma Radiometabolite Analysis

**Figure 2:**
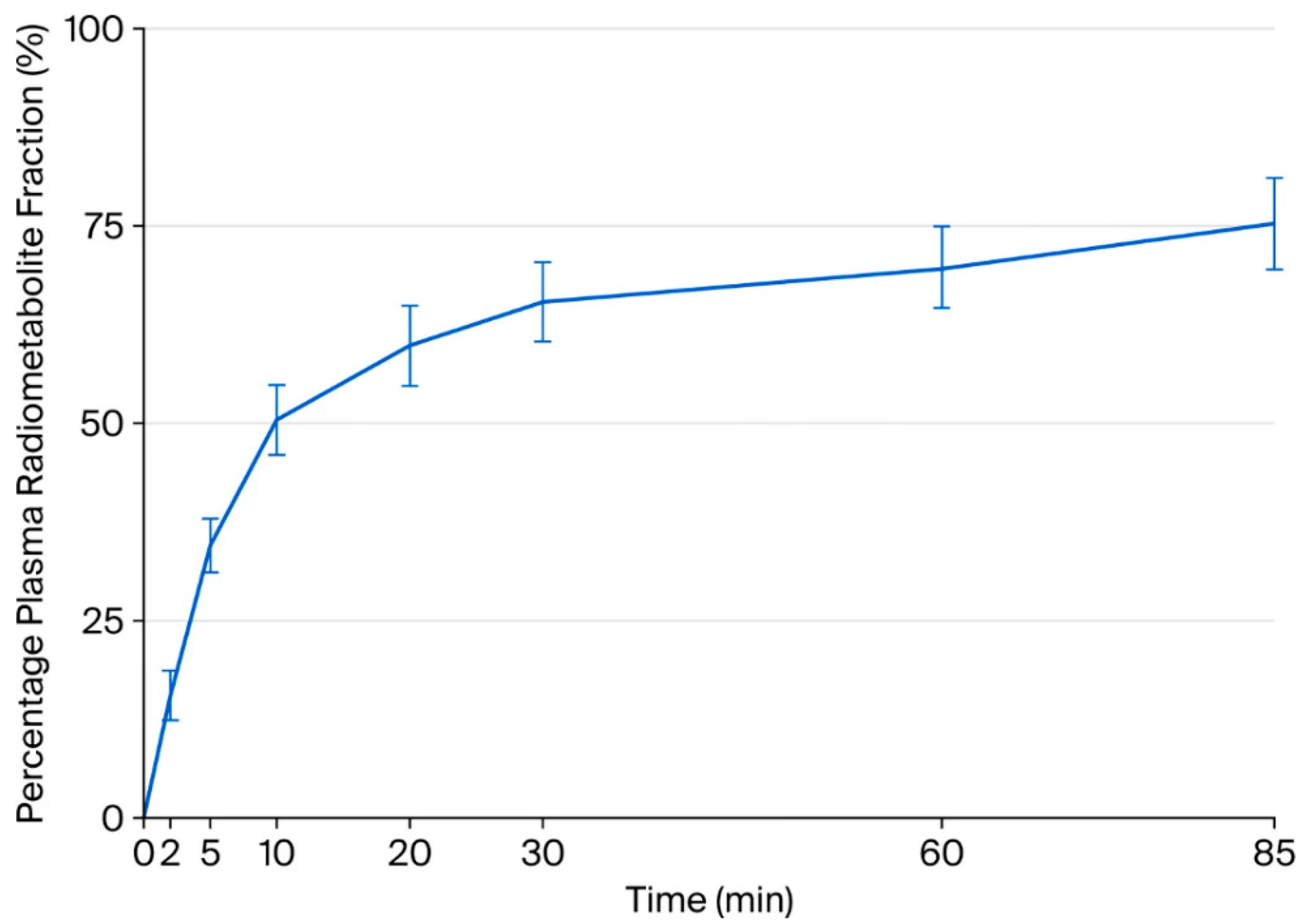
Percentage plasma radiometabolite fraction over time (mean ± SE, *n* = 5). Total radiometabolites rose to 50% by 10 min and 75% by 85 min post-injection.

**Figure 3:**
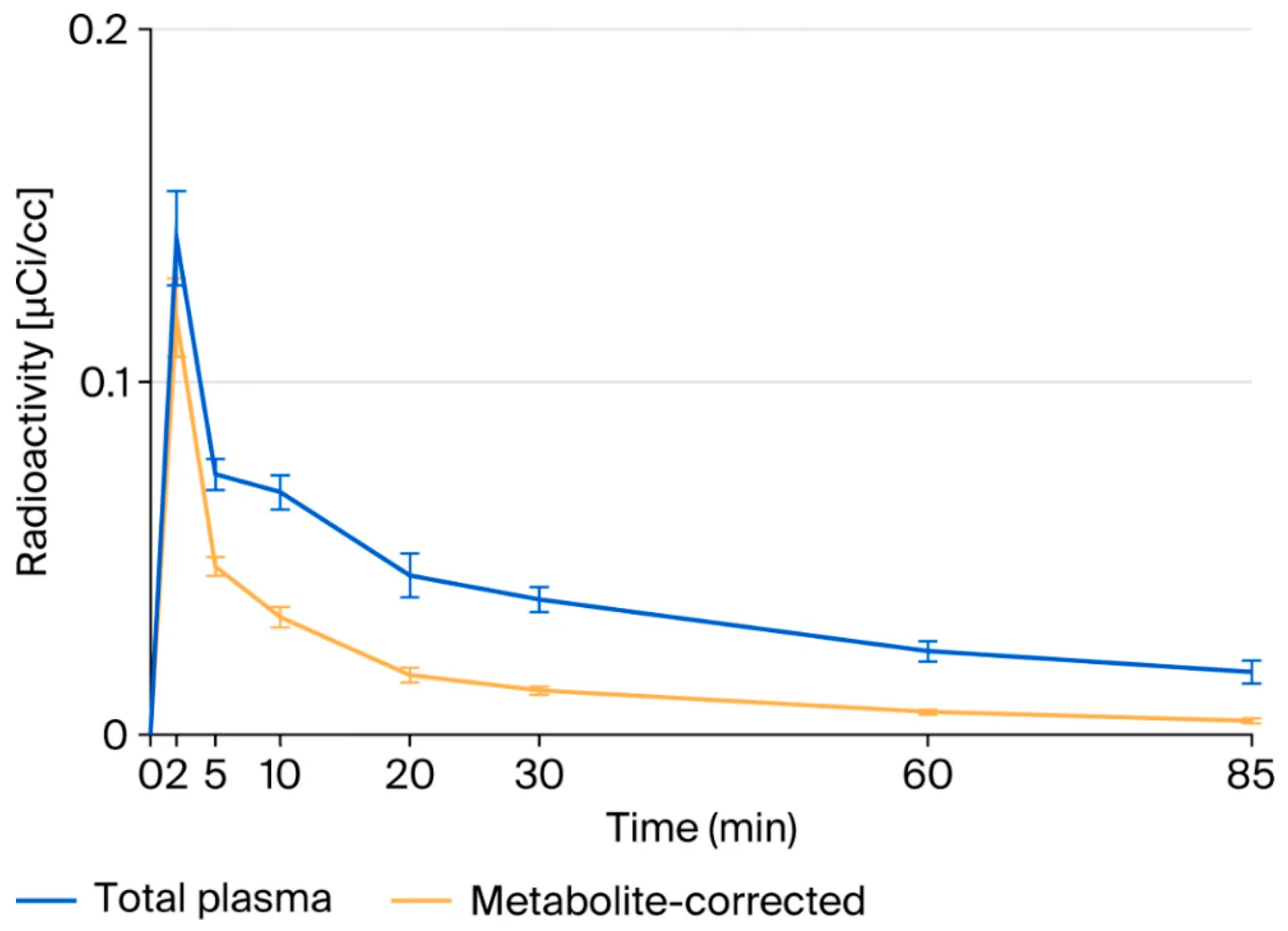
Total and metabolite-corrected plasma time–activity curves (mean ± SE, *n* = 5). Metabolite-corrected plasma activity showed a rapid decrease at 2 min and continued to decrease to 25% by 20 min.

**Figure 4:**
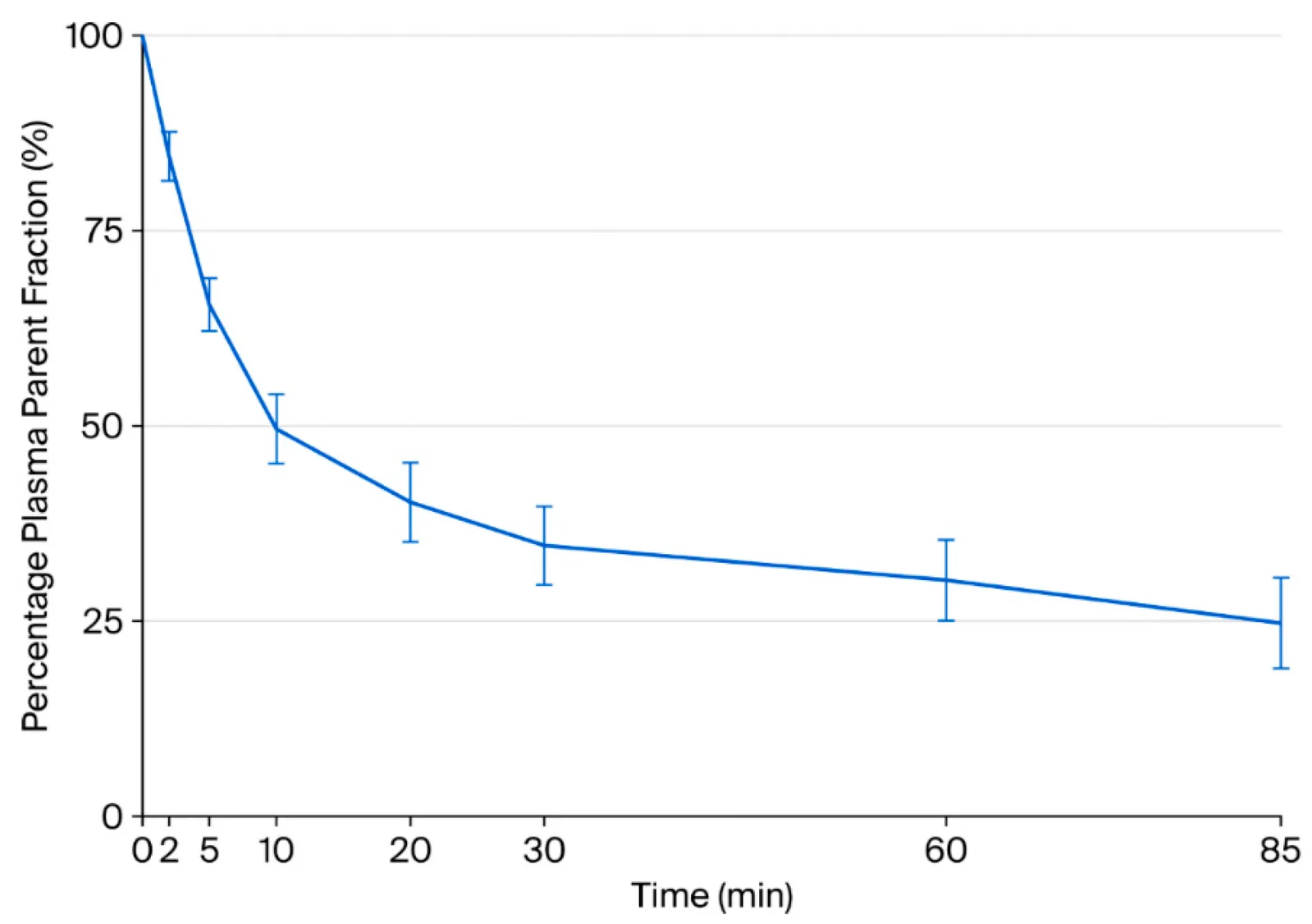
Derived percentage plasma parent fraction over time (mean ± SE, *n* = 5). Plasma parent fraction decreased from 84 ± 9% at 2 min to 40 ± 14% at 20 min and finally to 25 ± 15% at 85 min.

Radio-HPLC analysis consistently demonstrated two polar radiometabolite peaks eluting at 1.6 and 5.5 min, respectively, across all subjects with a radio-HPLC method. The percentage of plasma radiometabolite fraction increased progressively over time, accounting for approximately 50% of total plasma radioactivity at 10 min and 75% at 85 min post-injection. The chemical identities of these radiometabolites were not investigated in the present study. Correspondingly, the metabolite-corrected plasma parent fraction decreased rapidly from 84 ± 9% at 2 min to 40 ± 14% at 20 min and 25 ± 15% at 85 min post-injection (Figure 2, Figure 3 and Figure 4).

#### 2.2.2. Regional Brain Time–Activity Curves

Regional brain time–activity curves (TACs) demonstrated rapid tracer uptake, with peak activity occurring within approximately 5 min after injection. Regional differences in tracer retention were observed thereafter.

**Figure 5:**
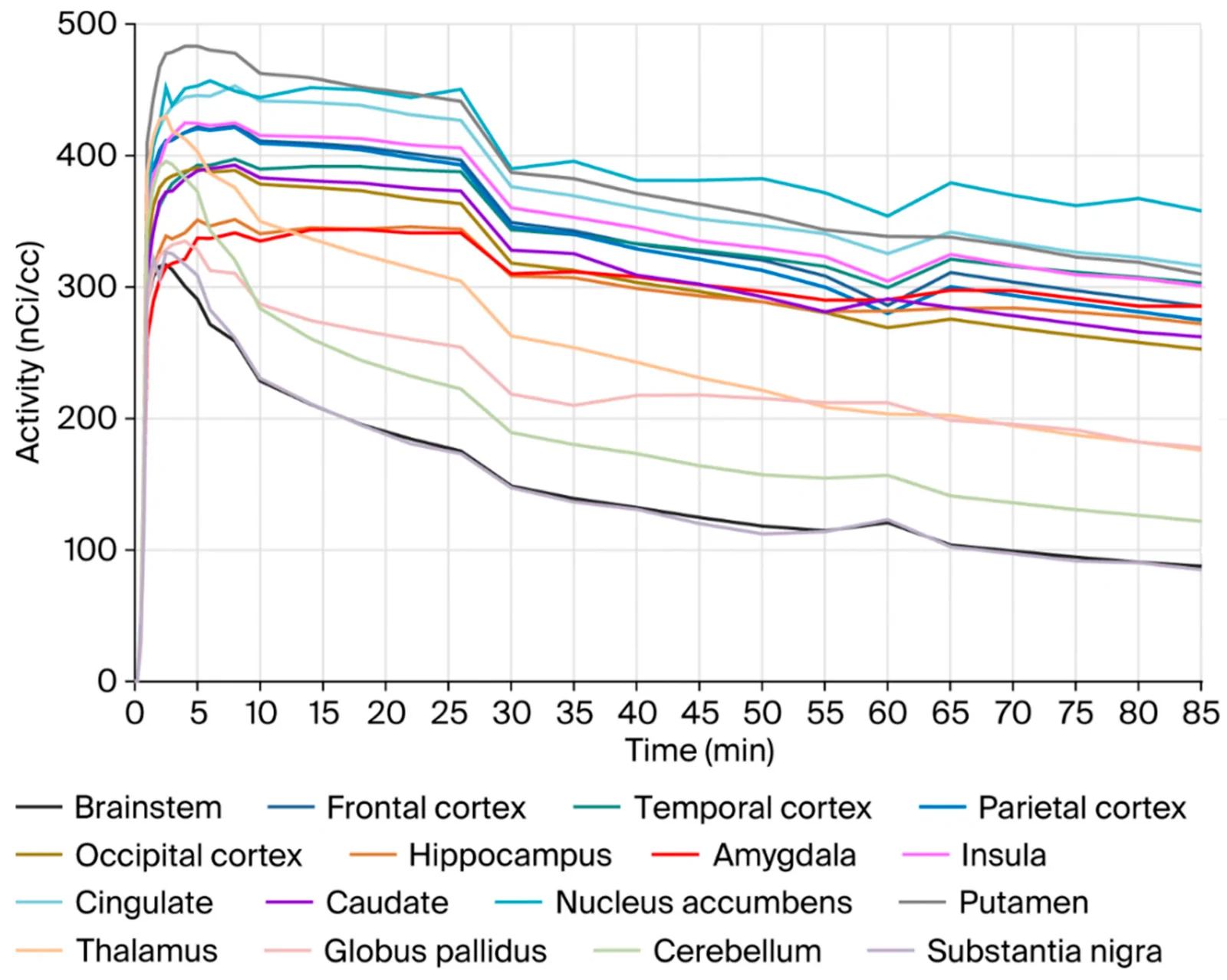
Decay-corrected brain regional mean TACs over time. The highest uptake and retention were observed in the nucleus accumbens, followed by the cingulate, putamen and insula; low tracer retention was seen in the brainstem and cerebellum.

**Figure 6:**
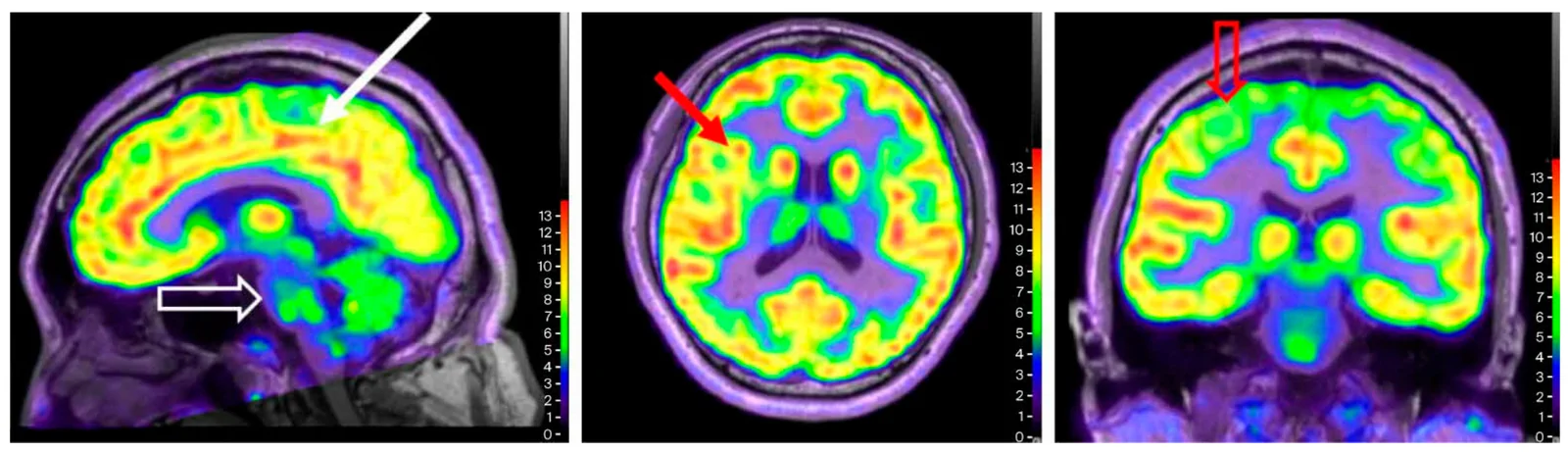
Representative multiplanar fused [18F]FPEB PET/MR images of a healthy subject displayed in sagittal (left figure), transverse (middle figure), and coronal orientations (right figure) Images represent the summed PET activity acquired from 60 to 85 min after tracer injection. Increased tracer uptake is observed in the cingulate gyrus (white solid arrow) and insula (red solid arrow), with moderate uptake in the frontal cortex (red hollow arrow) and minimal uptake in the pons (white hollow arrow) and cerebellum. The color scale indicates the relative regional [^18^F]FPEB uptake. All panels use the same color lookup table and intensity scale; differences in the displayed color bar length are due to the figure layout only. Representative sagittal, transverse, and coronal slices were selected independently to best illustrate the annotated brain regions.

The putamen exhibited the highest early tracer uptake immediately after injection, whereas the nucleus accumbens demonstrated the greatest tracer retention at later time points (from approximately 20 min onwards). Overall, the nucleus accumbens, cingulate gyrus, putamen, and insula showed the highest levels of [^18^F]FPEB uptake during the imaging period. Intermediate retention was observed in the thalamus and globus pallidus, whereas the lowest retention was observed in the brainstem and cerebellum (Figure 5 and Figure 6; Supplementary Table S1).

#### 2.2.3. Kinetic Model Evaluation

Model comparison using the Akaike Information Criterion (AIC) demonstrated that the two-tissue compartment model (2TCM) consistently provided a better fit to regional TACs than Logan graphical analysis across all brain regions and study scans.

Among the reference tissue models, SRTM and SRTM2 demonstrated comparable AIC values and generally outperformed MRTM and MRTM2 across most brain regions and subjects. Consequently, SRTM/SRTM2 were selected for subsequent reference tissue-based estimation of BPND.

#### 2.2.4. Regional Distribution Volume and Binding Potential

**Figure 7:**
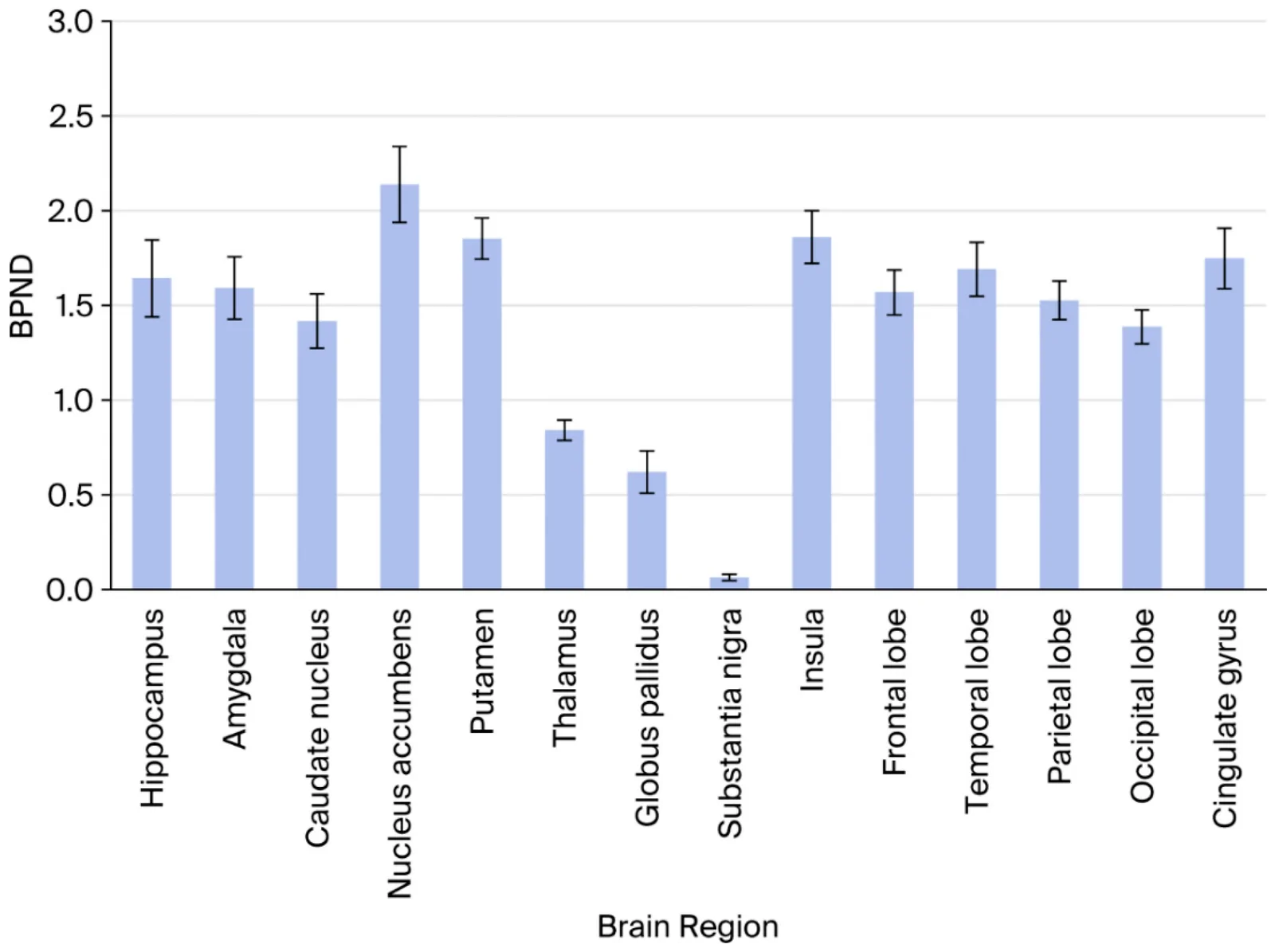
BPND per brain region using SRTM/SRTM2 (mean ± SE, *n* = 5). The nucleus accumbens had the highest BPND values, followed by the putamen, insula and cingulate gyrus.

**Figure 8:**
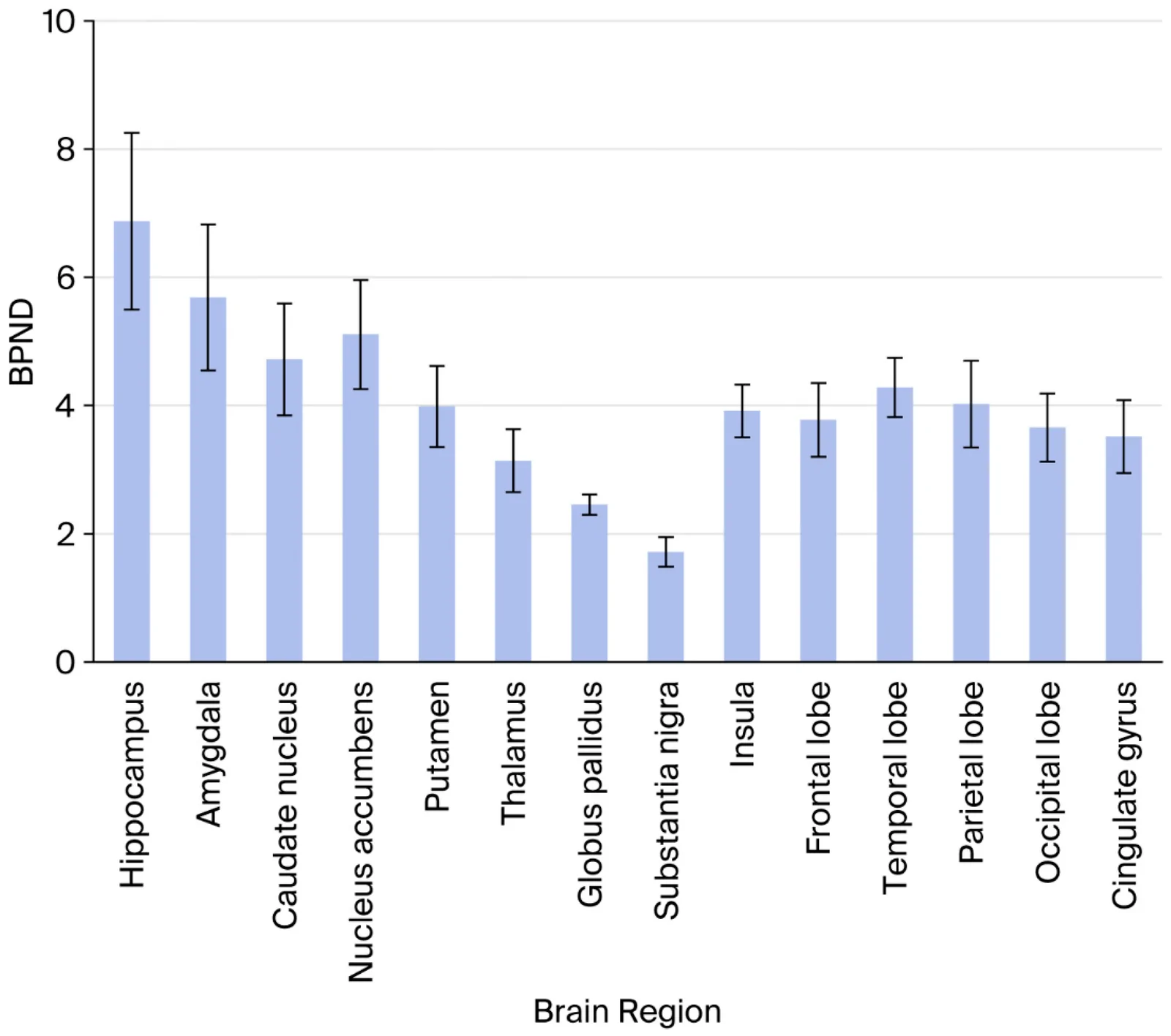
BPND per brain region using 2TCM-C (mean ± SE, *n* = 5). The hippocampus had the highest BPND values, followed by the amygdala, nucleus accumbens and caudate nucleus.

**Figure 9:**
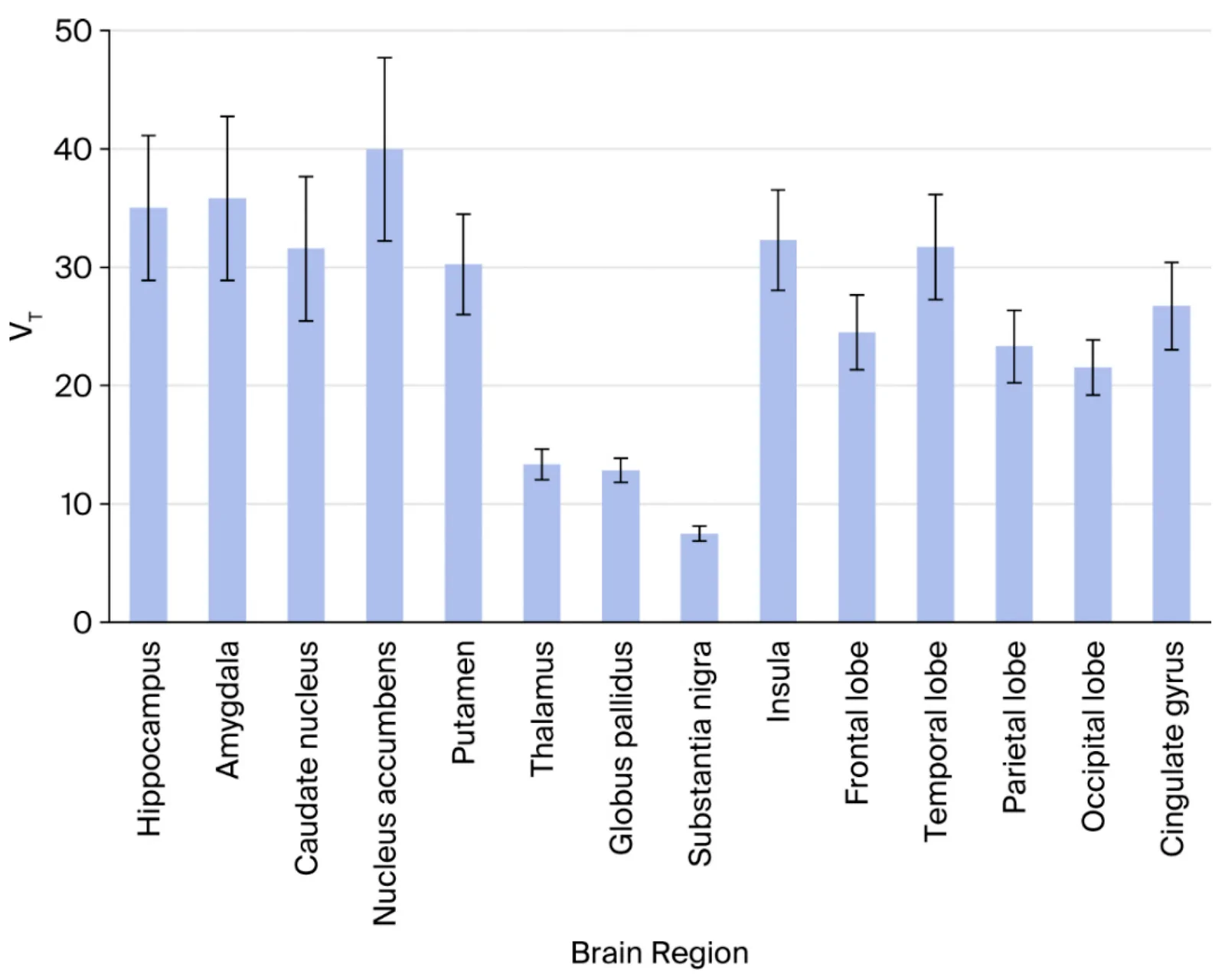
V_T_ per brain region using 2TCM-C (mean ± SE, *n* = 5). Similar to BPND values, the highest values were observed in the nucleus accumbens, amygdala and hippocampus.

Amongst the reference tissue models, SRTM/SRTM2 was used to calculate BPND values as it was the predominant best-fit model. The highest BPND values were seen in the nucleus accumbens, followed by the putamen, insula and cingulate gyrus (Figure 7). Amongst the blood-based models, tissue compartment models were used to calculate V_T_ and BPND values as they demonstrated a better fit based on AIC values. In particular, 2TCM-C was used over 2TCM in view of prior studies demonstrating more stable estimates at later timepoints [22]. The highest BPND values were seen in the hippocampus, followed by the amygdala, nucleus accumbens and caudate nucleus (Figure 8). V_T_ reflected a similar trend, with the highest values seen in the nucleus accumbens, amygdala and hippocampus (Figure 9).

To further explore the relationship between reference tissue and blood-based kinetic modeling, a Spearman correlation analysis was performed between SRTM/SRTM2-derived and 2TCM-C-derived BPND values averaged across subjects. A statistically significant, moderate positive correlation was observed (Spearman’s rs = 0.56, *p* = 0.042; Supplementary Figure S1). Given the exploratory nature of this study and the limited sample size, these findings should be interpreted with caution and require validation in larger cohorts.

#### 2.2.5. Test–Retest Reproducibility

Test–retest analyses were completed in three subjects because two participants did not undergo repeat imaging owing to subject withdrawal and technical issues. Retest scans were performed 24 ± 8 days after the initial scan (range 14–35 d). For 2TCM-C, the mean test–retest variability was 43% for V_T_ and 44% for BPND across brain regions. For the reference tissue models (SRTM/SRTM2), the mean test–retest variability for BPND was 33%.

Overall, test–retest variability exceeded 10% for all kinetic outcome measures and brain regions examined. The relatively high test–retest variability observed in the present study may limit the ability to detect small changes in mGluR5 availability. Nevertheless, larger disease-related alterations or pharmacologically induced receptor occupancy effects may remain detectable. Future studies with larger cohorts will be required to establish the minimum detectable effect size and determine the suitability of [^18^F]FPEB PET for longitudinal and treatment-response studies.

## 3. Discussion

The whole-body dosimetry findings in the present study are consistent with those reported previously by Wong et al. [22] in a US cohort. In both studies, the gallbladder was identified as the critical organ receiving the highest absorbed radiation dose. The mean whole-body effective dose of [^18^F]FPEB observed in our study was comparable to that reported by Wong et al. and is within the range of radiation exposures commonly encountered in clinical PET imaging. Notably, the effective dose of [^18^F]FPEB is comparable to that of widely used PET radiotracers such as [^18^F]FDG [27]. Unlike [^18^F]FDG, for which the urinary bladder is typically the dose-limiting organ, the gallbladder was the critical organ for [^18^F]FPEB, likely reflecting its predominant hepatobiliary clearance pathway.

Our plasma temporal profiles of total radiometabolites, metabolite-corrected plasma activity and parent fractions were similar to those observed by Wong et al. [22].

The regional brain uptake and retention patterns observed in this Asian cohort were comparable to the previous studies performed in healthy US volunteer cohorts [21,22]. The highest tracer retention was observed in the basal ganglia and limbic regions, moderate retention was seen in the thalamus, and the lowest retention was observed in the brainstem and cerebellum. This distribution closely mirrors the known distribution of mGluR5 within the human brain, where receptor density is highest in limbic and basal ganglia structures and relatively low in the cerebellum and brainstem [28]. The observed uptake pattern therefore provides further support for the high specificity of [^18^F]FPEB for mGluR5. This specificity is particularly important given the distinct physiological roles and anatomical distributions of different mGluR subtypes [6,29], as well as the growing interest in mGluR5 as a therapeutic target in a variety of neuropsychiatric and neurodegenerative disorders [3,6,9,10,11,12,30].

To our knowledge, this is the first study to characterize the biodistribution, dosimetry and brain pharmacokinetics of [^18^F]FPEB in an Asian study cohort. The overall similarity of our findings to previously published data [21] suggests that the pharmacokinetic and imaging characteristics of [^18^F]FPEB are largely conserved across these cohorts. This study was designed as a preliminary methodological and dosimetry investigation rather than a population-based study. The primary objectives were to establish the biodistribution, radiation dosimetry, and kinetic characteristics of [^18^F]FPEB in healthy Asian study cohorts and to evaluate the feasibility of reference tissue modeling. These findings provide preliminary support for the broader applicability of mGluR5 PET imaging and mGluR5-targeted therapeutic development across different populations.

This is also the first study to directly compare blood-based kinetic models and reference tissue models for the quantification of [^18^F]FPEB in humans. The one-tissue compartment model was not evaluated because previous studies have consistently demonstrated its inferior performance relative to the two-tissue compartment model [21,22,31]. Logan graphical analysis was included because Wong et al. [22] suggested that it may provide estimates comparable to 2TCM, while reference tissue models were investigated based on preclinical findings by de Laat et al. [31], who reported that SRTM and MRTM produced BPND estimates similar to those derived from 2TCM.

Whereas previous human studies primarily focused on arterial-input kinetic modeling, the present study systematically evaluated both blood-based and reference tissue approaches. Although SRTM/SRTM2 and 2TCM/2TCM-C identified similar regions with high mGluR5 binding, differences were observed in both the absolute BPND values and regional ranking. SRTM/SRTM2 estimate BPND directly using a reference tissue without arterial blood sampling, whereas 2TCM/2TCM-C first estimate regional V_T_ from the arterial input function, from which BPND is subsequently derived using the cerebellar V_T_ as the reference. Consequently, systematic differences in absolute BPND values may occur between the two modeling approaches and should not be interpreted as disagreement in the overall regional distribution of mGluR5 binding. These findings are consistent with the observations of de Laat et al. [31] and suggest that reference tissue approaches may provide a viable alternative for estimating [^18^F]FPEB BPND in humans. The potential clinical significance of this observation is considerable. Unlike blood-based kinetic models, reference tissue methods do not require arterial cannulation, serial blood sampling, metabolite analysis or complex input function generation. Consequently, they may facilitate wider adoption of [^18^F]FPEB PET imaging in both research and clinical settings.

The kinetic model optimization performed in the present study was based on healthy subjects and may not fully reflect tracer behavior in pathological conditions. Disease-related alterations in receptor expression, cerebral perfusion, blood–brain barrier function, or non-specific binding could influence model performance. Consequently, although the present findings provide an important framework for quantitative [^18^F]FPEB PET imaging, further validation in relevant patient populations will be required before the proposed modeling approaches can be broadly applied in clinical studies.

Reliable non-invasive quantification of mGluR5 availability could be particularly valuable in the development of mGluR5-targeted therapeutics. PET-derived measures of receptor availability and target engagement may support model-informed dose selection, patient stratification and treatment monitoring [32,33]. Such approaches may be especially useful for compounds with narrow therapeutic windows or substantial inter-individual variability in treatment response [10,11,12].

Several limitations should be acknowledged. First, test–retest variability was relatively high, exceeding 20% for both V_T_ and BPND estimates and being more pronounced for blood-based models than for reference tissue approaches. These values were higher than those reported by Wong et al. [22], who observed V_T_ variability of approximately 10–20% and BPND variability below 10% in most brain regions. One possible explanation is the longer interval between test and retest scans in the present study (14–35 days versus 2–16 days in Wong et al.). Additional sources of variability may include subject motion during image acquisition, technical variations between scans and inaccuracies in arterial blood sampling or metabolite correction procedures. These factors may also explain why variability was greater for blood-based models than for reference tissue methods.

Second, the sample size was limited, particularly for the test–retest analysis, because of subject withdrawal and technical challenges. Consequently, the reproducibility estimates reported here should be interpreted with caution and require confirmation in larger studies with repeated measurements.

Furthermore, only male subjects were included in this study. Potential sex-related differences in mGluR5 expression, receptor availability, or [^18^F]FPEB pharmacokinetics could not be evaluated and warrant investigation in future studies involving both male and female participants.

Despite these limitations, the present study provides preliminary evidence supporting the feasibility of reference tissue modeling for quantification of [^18^F]FPEB binding in the human brain. The practical challenges encountered during arterial blood sampling further highlight the potential advantages of non-invasive approaches. Continued development and validation of reliable reference tissue models may therefore enhance the clinical utility of mGluR5 PET imaging and facilitate future translational and therapeutic studies.

## 4. Materials and Methods

### 4.1. Radiotracer Synthesis and Quality Control

[^18^F]FPEB was synthesized on an automated GE Tracerlab FxFn synthesis module. Briefly, cyclotron-produced [^18^F]fluoride was trapped on a quaternary ammonium anion (QMA) cartridge, eluted with a Kryptofix 2.2.2/potassium carbonate solution, and azeotropically dried with acetonitrile under nitrogen. The iodonium ylide precursor for [^18^F]FPEB (Advanced Biochemical Compounds, Radeburg, Saxony, Germany), dissolved in dimethylformamide, was then added, and the reaction mixture was heated at 80 °C for 5 min. Upon completion of the radiolabeling reaction, the crude mixture was diluted with 5 mL H_2_O, and then the solution passed through a C18 SepPak cartridge and washed with 15 mL H_2_O. The product captured on the SepPak was eluted with 1 mL of EtOH, then 2 mL H_2_O was added. The resulting mixture was then loaded onto a semi-preparative HPLC column, Phenomenex Luna C18 column (250 × 10 mm, 10 μm, 100 Å), eluting with an isocratic method (EtOH:H_2_O = 60:40, *v*/*v*) at a flow rate of 5 mL. A typical chromatogram for the semi-preparative HPLC is shown in Figure S2. The product fraction (eluting at 18–21 min) was collected and diluted with 15 mL H_2_O in a round-bottom flask. The diluted product solution was then passed through a second C18 SepPak cartridge and washed with 15 mL H_2_O. The final product was eluted from the second SepPak with 1 mL EtOH, followed by 3 mL saline into a product vial pre-loaded with 7 mL saline.

Radiochemical purity, chemical purity and specific activity of [^18^F]FPEB were determined by analytical HPLC analysis of the final product. The mobile phases consisted of (A) acetonitrile/water containing 5 mM PIC B7 (Waters, Milford, MA, USA), an ion-pairing reagent containing heptanesulfonate, in a 70:30 (*v*/*v*) ratio and (B) acetonitrile/water containing 5 mM PIC B7 in a 30:70 (*v*/*v*) ratio. A Phenomenex Luna C18 analytical column (250 × 4.6 mm, 5 μm, 100 Å) was used, and eluted with a gradient program: 10% A/90% B (0–4 min), 90% A/10% B (5–8 min), and followed by re-equilibration to 10% A/90% B (9–10 min) at a flow rate of 2.0 mL/min. Under these chromatographic conditions, [^18^F]FPEB was eluted with a retention time of 4.6 min. The identity of [^18^F]FPEB was confirmed by comparison of the retention time of analytical HPLC chromatograms for the FPEB reference standard, recorded with a UV detector, to the retention time of [^18^F]FPEB determined using UV and radiometric detectors (Figure S2). The radiosynthesis afforded the final radiopharmaceutical with high radiochemical purity (>98%), excellent decay-corrected radiochemical yield (75 ± 5%) at the end of synthesis and molar activity (65 ± 30 GBq/µmol) at the time of injection, demonstrating the robustness and reproducibility of the radiosynthetic procedure. A trace UV peak consistent with the precursor was observed in the final product chromatogram. The available data indicate that the precursor was present only at trace levels, consistent with the molar activity of the administered [^18^F]FPEB; however, the exact precursor content could not be retrospectively quantified because the original chromatographic integration data were unavailable.

### 4.2. Study Participants

Healthy volunteers were recruited through institutional advertisements and community outreach initiatives. Interested individuals underwent screening to determine eligibility according to the predefined inclusion and exclusion criteria. The study consisted of two independent parts. Part I evaluated whole-body biodistribution and radiation dosimetry, whereas Part II investigated brain pharmacokinetics and kinetic modeling of [^18^F]FPEB. Five healthy Asian male volunteers (mean age 38 ± 7 years, range 28–47 years body weight 74.7 ± 11.8 kg (range, 61.9–85.8 kg)) were recruited for each part of the study.

All participants underwent screening procedures including medical history, physical examination, complete blood count, serum biochemistry and urinalysis. Subjects with a history of neurological or psychiatric disorders, significant medical illness, head trauma, metallic implants, or recreational drug use (including barbiturates, benzodiazepines, amphetamines and cocaine) were excluded. To minimize physiological variability, participants were instructed to avoid alcohol and caffeine-containing beverages prior to each PET examination, and identical imaging, blood-sampling, and processing protocols were used for both test and retest studies.

Safety assessments, including laboratory investigations, were repeated within one week following PET imaging. Written informed consent was obtained from all participants. All subjects provided informed consent before participating in the study. The study was approved by the Centralised Institutional Review Board of Singapore (CIRB 2016/2080).

### 4.3. PET/CT Imaging

PET/CT imaging was performed using a Discovery 690 PET/CT scanner (GE Healthcare, Princeton, NJ, USA) with a transaxial spatial resolution of 5.09 mm full-width at half-maximum (FWHM) at 10 cm and an axial field of view of 15.7 cm. PET data were reconstructed into a 128 × 128 matrix using an ordered subset expectation maximization algorithm with CT-based attenuation and scatter correction.

#### 4.3.1. Whole-Body Biodistribution and Dosimetry

For Part I, a low-dose CT scan was acquired for attenuation correction, and [^18^F]FPEB (4.5 ± 0.5 mCi) was administered as a rapid intravenous bolus via the antecubital vein of the contralateral arm, followed immediately by a 10–20 mL normal saline flush. Four sequential whole-body PET scans were acquired at approximately 0, 10, 30 and 60 min after injection, resulting in a total imaging duration of approximately 100 min.

Twelve source organs were identified for dosimetry analysis. Regions of interest (ROIs) were manually delineated on PET images, and time–activity curves (TACs) were generated for each organ. Source organ residence times were calculated by fitting TACs using mono-exponential uptake and bi-exponential clearance functions. Residence times were obtained by integration of the fitted curves and normalization to the administered activity.

Radiation absorbed doses and effective doses were estimated using the Medical Internal Radiation Dose (MIRD) schema implemented in OLINDA/EXM version 2.1.1 software [26].

#### 4.3.2. Brain Pharmacokinetic Imaging

In Part II, each subject underwent two dynamic [^18^F]FPEB PET scans using a test–retest design. The two imaging sessions were performed on separate days, with each scan lasting 85 min. Subjects were positioned using an individually fitted thermoplastic mask to minimize head movement. Following a low-dose CT scan for attenuation correction, [^18^F]FPEB (4.5 ± 0.5 mCi) was administered as a rapid intravenous bolus via the antecubital vein of the contra-lateral arm over 2 min, followed immediately by a 10–20 mL normal saline flush. Dynamic PET data were subsequently acquired in three-dimensional list mode for 85 min.

### 4.4. MRI Acquisition and Region-of-Interest Definition

All Part II participants underwent anatomical brain magnetic resonance imaging (MRI) prior to PET imaging. Magnetic resonance imaging was performed on a 3-T Siemens MRI system. A three-dimensional T_1_-weighted magnetization-prepared rapid gradient-echo (MPRAGE) sequence was acquired for anatomical localization and image co-registration. The imaging parameters were as follows: repetition time (TR), 2300 ms; echo time (TE), 2.98 ms; inversion time (TI), 900 ms; flip angle, 9°; field of view, 256 × 256 mm^2^; acquisition matrix, 256 × 256; 176 sagittal slices; slice thickness, 1.0 mm; and isotropic voxel size, 1.0 × 1.0 × 1.0 mm^3^. The acquisition time for the MPRAGE sequence was 5 min 12 s. MRI datasets were co-registered to a standard brain atlas using PMOD software (version 3.9; PMOD Technologies LLC, Zurich, Switzerland). Multiplanar fused PET/MR images (axial, coronal, and sagittal) were generated using the manufacturer’s software for anatomical localization of PET findings.

Twelve predefined ROIs were delineated, including the frontal, temporal, parietal and occipital cortices, caudate nucleus, putamen, amygdala, hippocampus, thalamus, cingulate gyrus, pons and cerebellum. Co-registration of MRI and PET datasets enabled accurate anatomical localization and extraction of regional PET time–activity curves. Only a single MRI examination was performed for each subject.

### 4.5. Arterial Blood Sampling and Metabolite Analysis

Arterial blood radioactivity concentrations were measured using an automated gamma counter (PerkinElmer Wizard2^®^, Waltham, MA, USA). All measurements were corrected for radioactive decay and background activity.

For metabolite analysis, arterial blood samples were collected from a radial arterial catheter at 0, 2, 5, 10, 20, 30, 60, and 90 min after intravenous administration of [^18^F]FPEB. Plasma was separated by centrifugation, and a 1 mL plasma aliquot was mixed with an equal volume (1 mL) of acetonitrile (1:1, *v*/*v*) for protein precipitation. Following vortex mixing, the samples were centrifuged at 10,000× *g* for 90 s, and the resulting supernatant was passed through a 0.22 μm polyvinylidene fluoride (PVDF) syringe filter before analysis by reverse-phase radio-HPLC using a Phenomenex Luna C18 column (150 × 4.6 mm, 3 μm, 100 Å; Phenomenex, Torrance, CA, USA), as previously described [34]. Chromatographic separation was performed using gradient elution with acetonitrile containing 0.1% formic acid and water containing 0.1% formic acid at a flow rate of 1.0 mL/min. The parent fraction of [^18^F]FPEB was calculated at each time point and used to generate metabolite-corrected plasma input functions for kinetic modeling.

### 4.6. Kinetic Modeling

Kinetic analyses were performed using PMOD software (version 3.9; PMOD Technologies LLC, Zurich, Switzerland). Regional TACs were extracted from all ROIs and analyzed using both plasma input and reference tissue models.

Plasma input models included the two-tissue compartment model (2TCM), constrained two-tissue compartment model (2TCM-C), and Logan graphical analysis [35]. For 2TCM-C, *V_T_* of the cerebellum was calculated using the formula $V_{T}=\frac{K_{1}}{k_{2}}\left(1+\frac{k_{3}}{k_{4}}\right)$, which assumes the cerebellum contained negligible or zero specific receptor binding (i.e., k_3_/k_4_ = 0); the *K1*/*k2* ratio was constrained using the cerebellar estimate to improve parameter identifiability. The mean *V_T_* of the cerebellum across subjects is 0.68 ± 0.08 (mean ± SE, *n* = 5). For the compartmental models, regional total distribution volumes (*V_T_*) were estimated using PMOD software. Binding potential relative to the non-displaceable compartment (*BPND*) was subsequently derived from the regional *V_T_* values using the cerebellum as the reference region according to: $BPND=V_{T,ROI}/V_{T,Ref}-1$, where V_T,ROI_ represents the total distribution volume of the region of interest, and V_T,ref_ is the total distribution volume of the cerebellum, which was assumed to represent the nondisplaceable distribution volume. Reference tissue approaches included the Simplified Reference Tissue Model (SRTM) [36], Simplified Reference Tissue Model 2 (SRTM2) [37], Multilinear Reference Tissue Model (MRTM) [38], and Multilinear Reference Tissue Model 2 (MRTM2) [39]. The brainstem was selected as the reference region for SRTM/SRTM2 analyses based on its low tracer uptake and previously reported negligible specific mGluR5 binding [22,34]. As required by the model assumptions, the reference region was assumed to have negligible specific mGluR5 binding (BPND ≈ 0); therefore, no BPND value was estimated for the brainstem itself. For plasma input models, arterial input functions were corrected for radiometabolites and radioactive decay prior to model fitting. Model performance was evaluated using the Akaike Information Criterion (AIC) [40], with lower AIC values indicating superior goodness-of-fit.

### 4.7. Test–Retest Analysis

The reproducibility of kinetic outcome measures was assessed using test–retest variability (TRV), calculated according to Wong et al. [22]:

TRV (%) = (1/n) Σ[|Testi − Retesti|/((Testi + Retesti)/2)] × 100

where n represents the number of subjects. TRV was calculated separately for VT and BPND estimates derived from each kinetic model.

## 5. Conclusions

This study provides the first characterization of the biodistribution, radiation dosimetry, and brain pharmacokinetics of [^18^F]FPEB in a healthy Asian study cohort. The dosimetry, radiometabolite profile, regional brain uptake pattern, and pharmacokinetic characteristics were generally consistent with those previously reported in healthy volunteer US cohorts. In addition, reference tissue models, particularly SRTM and SRTM2, produced BPND estimates comparable to blood-based kinetic models, supporting their feasibility as practical alternatives for BPND estimation, although further validation in larger cohorts remains necessary. These findings provide preliminary baseline data for healthy Asian study cohorts; however, validation in larger cohorts is warranted to confirm the pharmacokinetic characteristics and generalizability of the results.

## Figures and Tables

**Table 1:** [^18^F]FPEB residence times in target organs across five subjects, derived from whole-body PET (mean, SD).

Target Organ	Residence Times (h)						
	Subject 1	Subject 2	Subject 3	Subject 4	Subject 5	Mean	SD
Bone marrow	0.07	0.08	0.09	0.10	0.09	0.09	0.01
Brain	0.11	0.16	0.24	0.12	0.27	0.18	0.07
Heart	0.01	0.01	0.01	0.02	0.00	0.01	0.01
Gallbladder	0.10	0.07	0.09	0.10	0.07	0.09	0.02
Kidneys	0.02	0.02	0.02	0.02	0.01	0.02	0.00
Liver	0.12	0.39	0.27	0.38	0.15	0.26	0.13
Upper colon	0.01	0.01	0.01	0.01	0.00	0.01	0.00
Lower colon	0.01	0.01	0.01	0.01	0.01	0.01	0.00
Small bowel	0.41	0.34	0.34	0.39	0.39	0.37	0.03
Spleen	0.00	0.00	0.00	0.01	0.00	0.00	0.00
Thyroid	0.00	0.00	0.00	0.00	0.00	0.00	0.00
Urinary bladder	0.07	0.05	0.10	0.24	0.22	0.14	0.09
Residual body	1.05	1.05	0.76	1.19	1.19	1.05	0.18

[^18^F]FPEB: (^18^F)-3-fluoro-5-[(pyridin-3-yl)ethynyl] benzonitrile; PET: positron emission tomography; SD: standard deviation.

**Table 2:** Dosimetry results in target organs across five subjects, as calculated by OLINDA/EXM (mean, SD) [26], with the highest absorbed dose seen in the gallbladder.

Target Organ	Absorbed Dose (mGy/mCi)						
	Subject 1	Subject 2	Subject 3	Subject 4	Subject 5	Mean	SD
Bone marrow	0.53	0.54	0.49	0.54	0.59	0.54	0.04
Brain	1.6	1.64	0.75	0.7	1.19	1.18	0.45
Heart	0.28	0.41	0.39	0.58	0.5	0.43	0.11
Gallbladder	4.26	6.22	6.52	5.73	5.44	5.63	0.88
Kidneys	0.47	0.78	0.75	0.72	1.4	0.82	0.34
Liver	0.82	1.52	0.79	1.72	2.25	1.42	0.62
Upper colon	0.8	0.94	1.02	0.93	1.06	0.95	0.10
Lower colon	0.75	0.69	0.74	0.73	0.77	0.74	0.03
Small bowel	2.98	2.99	3.43	2.87	3.25	3.10	0.23
Spleen	0.29	0.26	0.29	0.52	0.36	0.34	0.11
Thyroid	0.25	0.2	0.23	0.22	0.26	0.23	0.02
Urinary bladder	3.89	2.15	1.58	3.99	1.35	2.59	1.27
Residual body	0.37	0.35	0.35	0.38	0.44	0.38	0.04
Testes	0.28	0.2	0.24	0.28	0.26	0.25	0.03
Effective dose	0.66	0.63	0.60	0.72	0.70	0.66	

SD: standard deviation.

## Data Availability

The original contributions presented in this study are included in the article/Supplementary Materials. Further inquiries can be directed to the corresponding authors.

## Supplementary Materials

The following supporting information can be downloaded at: https://www.mdpi.com/article/10.3390/ph19091375/s1. Figure S1. Spearman correlation of BPNDs between SRTM/SRTM2 and 2TCM-C; Figure S2. (Top) A typical semi-preparative HPLC chromatogram for [^18^F] FPEB; (Bottom) quality control analytic HPLC chromatographs confirmed the identity of [^18^F]FPEB; Table S1. Table of decay-corrected mean regional brain [^18^F]FPEB activity (nCi/cc) over time.
