# Supplementary material for: Biodistribution, Radiation Dosimetry, and Kinetic Modeling of [18F]FPEB PET Imaging of mGluR5 in Healthy Asian Subjects
Source: Pharmaceuticals (Basel). 2026 Aug 31;19(9):1375. doi: 10.3390/ph19091375 (PMC13610761; doi:10.3390/ph19091375)
Supplement: Supplementary file 1 [file pharmaceuticals-19-01375-s001.zip › pharmaceuticals-4384025-supplementary.pdf]

Supplementary Materials

For the manuscript "Biodistribution, Radiation Dosimetry, and Kinetic Modeling of [^{18}F]FPEB PET Imaging of mGluR5 in Healthy Asian Subjects"

Wanying Xie [†1,2]*, Sarah Beishan Tai [†1,2], Chang-Tong Yang [1,2]*, Sidney Yu [1,3], Young Soon Tay, Ruban Poopalalingam [1, 4], Darren Koh [4], Kerry Riffel [5], Wenping Li [5], Chih-Liang Chin[6], Abu Bakar Ali Asad [7], Darren Wan Teck Lim [8], David Chee Eng Ng [1,2]

† Denotes co-first authorship

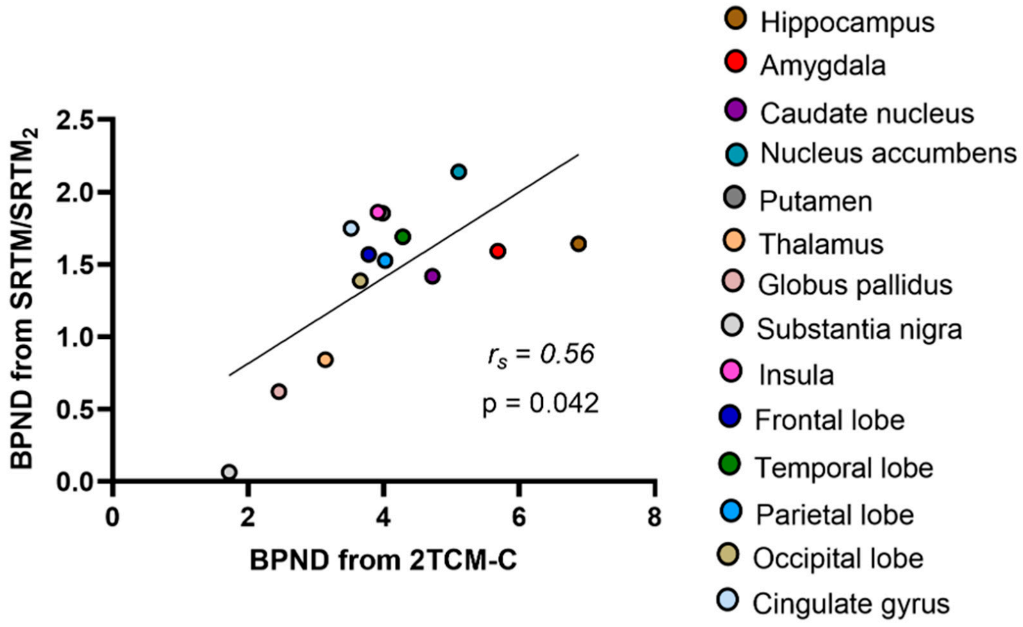

Figure S1. Spearman correlation of BPNDs between SRTM/SRTM2 and 2TCM-C.

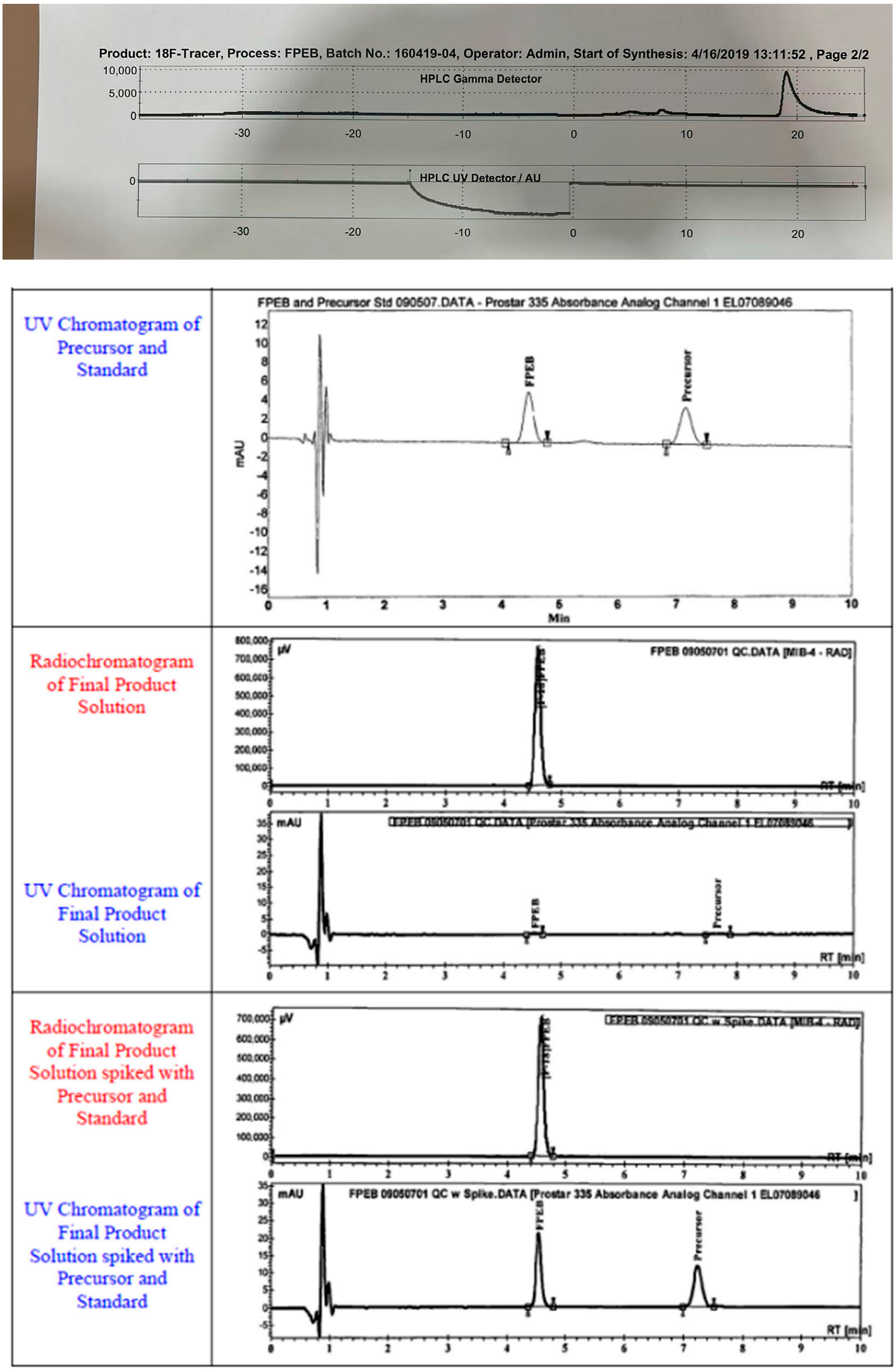

Figure S2. (Top) A typical semi-preparative HPLC chromatogram for [^{18}F] FPEB; (Bottom) quality control analytic HPLC chromatographs confirmed the identity of [^{18}F]FPEB.

The identity of [^{18}F]FPEB has been confirmed by the comparison of the retention time in analytical HPLC chromatograms for the FPEB reference standard, recorded with a UV detector, with the retention time of [^{18}F]FPEB determined using UV and radiometric detectors. When [^{18}F]FPEB was mixed with the FPEB Reference Standard and reanalyzed by HPLC, the UV absorbance and radioactivity retention times matched completely, with correction of the time off-set between the two detectors (Figure S2).

Table S1. Table of decay-corrected mean regional brain [^{18}F]FPEB activity (nCi/cc) over time.

nCi/cc																	
Time_Seconds	Time_Minutes	Brainstem	FrontalCor	TemporalC	ParietalCo	OccipitalC	Hippocam	Amygdala	Insula	Cingulate	Caudate	NucleusAc	Putamen	Thalamus	GlobusPall	Cerebellur	Substantia
0	0	0.073912	0.050809	0.064841	0.080765	0.059153	0.047084	0.050091	0.054925	0.051572	0.048902	0.026324	0.114588	0.075204	0.064212	0.033478	0.012467
15	0	0.526905	0.580765	0.557383	0.384494	0.342276	0.359057	1.331437	0.930432	0.614858	0.757566	0.454176	0.696339	0.399209	0.531903	0.499171	0.357915
30	1	36.03011	39.3369	35.70183	38.4911	34.70352	34.01484	36.04109	49.98365	48.66545	30.7922	48.15902	42.49698	39.9758	30.73532	34.45332	34.08667
45	1	137.2574	162.8084	146.5062	163.4318	148.2047	141.3601	132.7545	175.4931	183.6813	129.7095	174.8697	178.5655	171.9958	123.5984	153.1131	123.5205
60	1	286.7201	356.3452	317.4472	358.7707	331.3972	293.6453	260.1546	357.3349	378.1824	309.3678	364.5551	409.1176	386.4059	285.6124	343.735	299.2138
75	1																
90	2	308.2219	386.8821	344.6124	389.5729	362.0701	314.9972	289.6535	385.295	409.6827	342.5419	407.653	444.1118	414.6173	311.2561	376.5096	321.2449
105	2																
120	2	315.4506	401.597	361.7633	404.0839	375.4708	327.6346	305.0091	394.0569	420.9887	364.6214	424.4575	467.127	427.2765	323.6699	391.6809	306.3587
135	2																
150	3	316.7179	409.7916	370.9504	411.0793	381.7992	338.6807	315.2338	409.0671	430.7265	372.167	451.4191	477.2774	429.9354	328.2643	395.4703	326.3631
165	3																
180	3	313.0478	411.5771	378.3116	412.2813	384.4138	336.593	317.8189	415.037	437.265	372.7292	438.0222	478.3515	418.8096	332.0318	393.1121	325.4346
195	3																
210	4																
225	4																
240	4	300.3536	417.4986	386.5251	417.3211	387.8108	341.2141	320.9917	424.5262	444.1803	381.8947	450.9482	482.9725	412.5895	334.7739	382.5901	317.0955
255	4																
270	5																
285	5																
300	5	290.6643	421.6071	392.4918	420.4402	390.5156	350.8976	336.9898	424.241	445.6722	388.305	452.7168	482.8973	402.8947	327.7976	372.4436	308.744
315	5																
330	6																
345	6																
360	6	271.5963	419.4781	392.4194	418.9136	387.2041	346.4637	336.7832	422.5731	444.9206	389.7606	456.7369	480.0535	386.3335	312.5796	342.9745	282.6331
375	6																
390	7																
405	7																
420	7																
435	7																
450	8																
465	8																
480	8	258.8476	423.2934	397.0138	421.4266	388.584	351.3362	341.2038	424.6019	452.6982	392.5055	449.0005	477.8434	375.5507	310.2739	320.5622	261.0232

Patients’ radiation dose list:

Subject 1

Radiation Dosimetry of F18 FPEB

Patient's name: WWS Patient's IC/Passport#:Sxxxx088I

Scan Number: 2018RF0001 F-18 Infusion Activity: 5mCi

Gender (Male/Female): Male Date of Infusion: 08/01/2018

Cycle of Infusion: 1 Weight: 90

Height: 178cm

1 Table of dose-limiting organ doses (mGy).

		Weighted Dose Using Patient Weight		
Target Organ	Residence Time (hr)	From Olinda Absorbed Dose (mGy)	From Olinda Effective Dose (mSv)	For Subsequent Rx Absorbed Dose (mGy/mCi)
Bone Marrow	0.09	2.64	0.32	0.53
Brain	0.27	7.99	0.04	1.60
Heart	0.00	1.38	0.00	0.28
Gallbladder	0.07	21.31	0.00	4.26
Kidneys	0.01	2.34	0.01	0.47
Liver	0.15	4.09	0.20	0.82
Upper Colon	0.00	4.01	0.02	0.80
Lower Colon	0.01	3.74	0.45	0.75
Small Bowel	0.39	14.89	0.07	2.98
Spleen	0.00	1.43	0.01	0.29
Thyroid	0.00	1.25	0.06	0.25
Urinary Bladder	0.22	19.45	0.97	3.89
Residual Body	1.19	1.85	0.00	0.37
Testes		1.41	0.00	0.28
Effective Dose(ED)***	3.31/5 = 0.66			

Internal dosimetry based on serial whole-body images and using the Olinda-Oragan Level Internal Dose Assessment Code (copyright Vanderbilt University, 2003) Adult Female or Adult Male Olinda Exam Model.

Subject 2

Radiation Dosimetry of F18 FPEB

Patient's name: OCE

Patient's IC/Passport#:SXXXX321C

Scan Number: 2017RF003

F-18 Infusion Activity: 4.9mCi

Gender (Male/Female): Male

Date of Infusion: 21/11/2017

Cycle of Infusion: 1

Weight: 68

Height: 165cm

		Weighted Dose Using Patient Weight		
Target Organ	Residence Time (hr)	From Olinda Absorbed Dose (mGy)	From Olinda Effective Dose (mSv)	For Subsequent Rx Absorbed Dose (mGy/mCi)
Bone Marrow	0.09	2.63	0.32	0.54
Brain	0.24	8.04	0.02	1.64
Heart	0.01	1.99	0.00	0.41
Gallbladder	0.09	30.46	0.00	6.22
Kidneys	0.02	3.63	0.01	0.78
Liver	0.27	7.45	0.37	1.52
Upper Colon	0.01	4.60	0.01	0.94
Lower Colon	0.01	3.38	0.41	0.69
Small Bowel	0.34	14.63	0.37	2.99
Spleen	0.00	1.26	0.00	0.26
Thyroid	0.00	1.00	0.05	0.20
Urinary Bladder	0.10	10.52	0.53	2.15
Residual Body	0.76	1.73	0.00	0.35
Testes		1.00	0.00	0.20
Effective Dose(ED)***	3.08/4.9 = 0.63			

Subject 3

Radiation Dosimetry of F18 FPEB

Patient's name: NWK Patient's IC/Passport#:Sxxxx839C

Scan Number: F-18 Infusion Activity: 5mCi

Gender (Male/Female): Male Date of Infusion: 07/11/2017

Cycle of Infusion: 1 Weight: 71

Height: 174cm

2 Table of dose-limiting organ doses (mGy).

		Weighted Dose Using Patient Weight		
Target Organ	Residence Time (hr)	From Olinda Absorbed Dose (mGy)	From Olinda Effective Dose (mSv)	For Subsequent Rx Absorbed Dose (mGy/mCi)
Bone Marrow	0.07	2.46	0.29	0.49
Brain	0.11	3.77	0.01	0.75
Heart	0.01	1.94	0.00	0.39
Gallbladder	0.10	32.59	0.00	6.52
Kidneys	0.02	3.73	0.01	0.75
Liver	0.12	3.93	0.20	0.79
Upper Colon	0.01	5.11	0.01	1.02
Lower Colon	0.01	3.70	0.44	0.74
Small Bowel	0.41	17.13	0.41	3.43
Spleen	0.00	1.45	0.00	0.29
Thyroid	0.00	1.13	0.06	0.23
Urinary Bladder	0.07	7.92	0.40	1.58
Residual Body	1.05	1.73	0.00	0.35
Testes		1.19	0.00	0.24
Effective Dose(ED)***	2.98/5 = 0.60			

Internal dosimetry based on serial whole-body images and using the Olinda-Oragan Level Internal Dose Assessment Code (copyright Vanderbilt University, 2003) Adult Female or Adult Male Olinda Exam Model.

Subject 4

Radiation Dosimetry of F18 FPEB

Patient's name: CWH

Patient's IC/Passport#:SXXXX021F

Scan Number: 2017RF004

F-18 Infusion Activity: 4.7mCi

Gender (Male/Female): Male

Date of Infusion: 28/11/2017

Cycle of Infusion: 1

Weight: 94

Height: 183cm

3 Table of dose-limiting organ doses (mGy).

		Weighted Dose Using Patient Weight		
Target Organ	Residence Time (hr)	From Olinda Absorbed Dose (mGy)	From Olinda Effective Dose (mSv)	For Subsequent Rx Absorbed Dose (mGy/mCi)
Bone Marrow	0.10	2.54	0.31	0.54
Brain	0.12	3.30	0.02	0.70
Heart	0.02	2.71	0.00	0.58
Gallbladder	0.10	26.94	0.00	5.73
Kidneys	0.02	3.36	0.02	0.72
Liver	0.38	8.09	0.40	1.72
Upper Colon	0.01	4.39	0.02	0.93
Lower Colon	0.01	3.42	0.41	0.73
Small Bowel	0.39	13.47	0.07	2.87
Spleen	0.01	2.45	0.01	0.52
Thyroid	0.00	1.05	0.05	0.22
Urinary Bladder	0.24	18.75	0.94	3.99
Residual Body	1.19	1.80	0.00	0.38
Testes		1.30	0.00	0.28
Effective Dose(ED)***	3.36/4.7 =0.715			

Internal dosimetry based on serial whole-body images and using the Olinda-Oragan Level Internal Dose Assessment Code (copyright Vanderbilt University, 2003) Adult Female or Adult Male Olinda Exam Model.

Subject 5

Radiation Dosimetry of F18 FPEB

Patient's name: BWC Patient's IC/Passport#:Gxxxx900U

Scan Number: F-18 Infusion Activity: 5mCi

Gender (Male/Female): Male Date of Infusion: 14/11/2017

Cycle of Infusion: 1 Weight: 64

Height: 162cm

4 Table of dose-limiting organ doses (mGy).

		Weighted Dose Using Patient Weight		
Target Organ	Residence Time (hr)	From Olinda Absorbed Dose (mGy)	From Olinda Effective Dose (mSv)	For Subsequent Rx Absorbed Dose (mGy/mCi)
Bone Marrow	0.08	2.97	0.36	0.59
Brain	0.16	5.97	0.01	1.19
Heart	0.01	2.48	0.00	0.50
Gallbladder	0.07	27.18	0.00	5.44
Kidneys	0.02	7.00	0.02	1.40
Liver	0.39	11.27	0.56	2.25
Upper Colon	0.01	5.32	0.01	1.06
Lower Colon	0.01	3.86	0.46	0.77
Small Bowel	0.34	16.24	0.40	3.25
Spleen	0.00	1.78	0.00	0.36
Thyroid	0.00	1.32	0.07	0.26
Urinary Bladder	0.05	6.73	0.33	1.35
Residual Body	1.05	2.19	0.00	0.44
Effective Dose(ED)***	3.49/5 = 0.698			

Internal dosimetry based on serial whole-body images and using the Olinda-Oragan Level Internal Dose Assessment Code (copyright Vanderbilt University, 2003) Adult Female or Adult Male Olinda Exam Model.
